# A critical appraisal of “effects of exercise therapy on disability, mobility, and quality of life in the elderly with chronic low back pain: a systematic review and meta-analysis of randomised controlled trials”

**DOI:** 10.1186/s13018-024-04766-0

**Published:** 2024-07-26

**Authors:** Pierre Novert, Mathilde Pelletier-Visa, Bruno Pereira, Charlotte Lanhers, Emmanuel Coudeyre

**Affiliations:** 1 Service de Médecine Physique et de Réadaptation, Centre Hospitalier Universitaire de Clermont-Ferrand, CHU Clermont-Ferrand, INRAE, UNH, Clermont- Ferrand, Clermont-Ferrand, F-63000 France; 2 Direction de la Recherche Clinique et de l’Innovation, secteur Biométrie-Economie, CHU de Clermont-Ferrand, 5 place Henri Dunant, Clermont-Ferrand, France

**Keywords:** Chronic low back pain, Physical exercise, Aged, Randomised controlled trial, meta-analysis

## Dear editor

Chronic low back pain (CLBP), defined as pain in the lumbar region lasting more than 3 months, is a major public health issue [1]. Optimal management strategies for this condition have yet to be clearly defined. Neither affected individuals or healthcare professionals are not satisfied with the usual treatments of analgesics, physical exercise and physiotherapy. We have very few studies on the therapeutic and physical management of low-back pain in populations aged over 55 on Pubmed.

Before starting, a reminder of some definitions. Exercise therapy [2] is a regimen or plan of physical activities designed and prescribed for specific therapeutic goals. Its purpose is to restore normal musculoskeletal function or to reduce pain caused by diseases or injuries.

Then, the definition of exercise [3] is a physical activity which is usually regular and done with the intention of improving or maintaining physical fitness or health. Contrast with physical exertion which is concerned largely with the physiologic and metabolic response to energy expenditure.

Exercise therapy is recommended as part of the treatment of individuals with CLBP [4]. Many studies have shown the value of exercise therapy in reducing pain and increasing function in people with CLBP [5].

Chronic low-back pain, requires answers to its management, and in particular whether exercise as a treatment for adults aged more than 55 years old with low-back pain is a reality or not [6].

As early as 2016, papers started but never finished in the Cochrane database. A motivated team has already published a meta-analysis on the same subject in 2022, with no age limit [7]. This proves the determination to respond to this epidemiological problem. A steadily increasing number of elderly patients with low back pain; many results on therapeutic exercises; but few publications on the aged patient, a major contrast.

Therefore, the study by Shi-kun Zhang et al. ***“Effects of exercise therapy on disability, mobility, and quality of life in the elderly with chronic low back pain: a systematic review and meta-analysis of randomised controlled trials”*** published in July 2023, is important as it provides specific answers regarding exercise therapy for individuals aged > 55 years [6]. Theauthors highlight exercises that can be used to update current programmes in line with recent recommendations [4], thus helping to bring about changes in current practice.

Although we commend the authors for this study, which involved a substantial amount of work and time, we wish to highlight 2 main issues that we believe severely impact its results.

### The first issue relates to the primary and secondary endpoints of the study

Which outcomes were primary and which were secondary is unclear. The authors define 2 primary outcomes: pain and disability. Pain assessed by Visual Analogic Scale (VAS). However, the title of the article mentions disability and mobility but not pain. Also, pain is not mentioned in the study hypotheses or at the beginning of the method where the other outcomes are listed. Furthermore the endpoints stated as secondary appear to have been analysed as primary endpoints, which is not in line with the Cochrane database recommendations [8]. In that article presents the general characteristics of the included studies ; only 8 out of 16 used a VAS rating of pain as the primary endpoint. In 5 studies, the primary endpoint was either the Timed Up and Go (TUG) or the Short Form 36 (SF36).

### The second issue is the risk of bias in the articles included in the meta-analysis

The authors had the patience to search for a number of studies on the subject of chronic low back pain and its management by exercise in the elderly which is a very time-consuming task. The authors had the wisdom to use a study quality assessment scale to clarify their results.

The meta-analysis shows bias in article selection, mainly favoring studies with high bias risk. Only 2 studies with low bias risk were included. Thus, concluding that exercise significantly reduces VAS in elderly low-back pain patients is challenging. The authors used the Revised Cochrane Collaboration Risk of Bias Tool for Randomised Trials (RoB2) to evaluate the quality of included RCT. Although if this tool is relevant, tools that are more specific for non-pharmacological [9], studies might have been considered. For example, the Downs and Black scale [10] has more criteria than the RoB2 and, therefore, the risk of study rejection is lower. A good practice is to exclude the high-risk studies from the analysis of the primary endpoint as they could alter the results. In our opinion, the inclusion of a majority of studies at high bias risk, with only 2 at low risk of bias, makes it difficult to conclude as to a significant effect of exercise on reducing the VAS rating of pain in older people with low back pain.

**Fig. 1:**
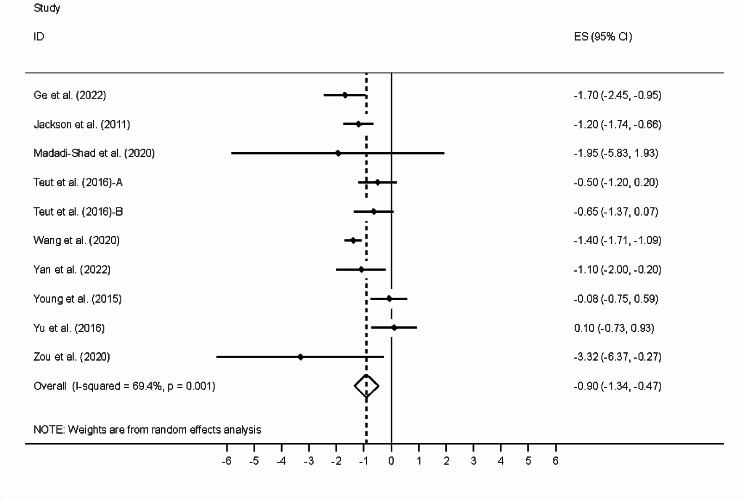
Meta-analysis of the effects of different forms of exercise therapy on VAS pain rating. Results show the weighted mean with the 95% confidence interval

We verified if the results were different without the studies at high risk of bias. Figure 1 shows the sensitivity analysis that we conducted. We excluded studies according to (i) their risk of bias, (ii) a funnel-plot analysis, and (iii) the results of Egger’s test. This resulted in the exclusion of the studies by Yalfany et al. [11] and Park et al. [12]. These studies were excluded one at a time in the study by Zhang et al., but they were not excluded together in that study.

The removal of the most biased of the 2 studies yields a weighted mean difference that is half of that found by Zheng et al.: 0.9 95% CI [0.47; 1.34] versus 1.75 95% CI [0.92; 2.59]. Furthermore, I², a measure of heterogeneity, decreased from 93 to 69.4% after the exclusion of the 2 studies with the highest level of bias.

Our reading of the article by Zhang et al. found a lack of precision and consistency across all criteria for the analysis. The use of a different sensitivity analysis grid would have improved precision by excluding poor-quality studies for the analysis of both the primary and secondary endpoints. Additionally, a complementary analysis of the same sample of studies for both endpoints would have been beneficial. Drawing conclusions from studies at high risk of bias can result in misleading findings.

Understanding of the results of the risk of bias analysis by Zheng et al. is limited by the lack of definition of the symbol ‘!’ present in this article. We were unable to find such a symbol in the Cochrane version of the sensitivity analysis grid and conclude that it is likely a typographical error.

We also found discrepancies in the flowchart shown in Fig. 1. The number of full texts.

assessed for eligibility is 183 on the flowchart, whereas, the number of records reviewed minus the number excluded yields 185. The sum of all the excluded studies listed in the figure is 164, not 168 as indicated. These discrepancies in the flowchart reduce our confidence in the meta-analysis performed.

Studies on CLBP in older adults are lacking, particularly in comparison to the abundance of research focusing on younger populations. The Cochrane database includes articles on exercise interventions such as Pilates or general physical activity for CLBP, however most studies predominantly involve individuals under 55 years old. To enhance precision, revisiting article databases before the selection deadline could allow for the addition of relevant studies and the removal of less impactful ones.

The review by Zhang et al. included studies with significant variability within the exercise and control groups, making comparisons challenging. To enhance clarity, the exercise and control therapies could have been categorized into aerobic exercises, core stability exercises, stretching, low-impact physical activities (like Pilates, yoga, Qigong, Baduanjin and Tai chi), and water sports.

Lastly, it seems important to state that the meta-analysis on Prospero (https://www.crd.york.ac.uk/prospero/) was registered a posteriori, on May 31, 2023, whereas the publication was submitted on February 25, 2023, which does not comply with article writing rules.

CLBP is a significant public health issue, especially in older individuals. The effectiveness of physical therapy and exercise, which is the recommended treatment for adults aged 18–55 years, in managing this condition among the elderly is still uncertain. Clear and precise answers are eagerly awaited by both affected individuals and healthcare providers. Reliable, high-quality analyses on a larger scale are crucial. Future research should focus on refining selection criteria and using a more comprehensive quality assessment grid for articles included in meta-analyses.

## Data Availability

No datasets were generated or analysed during the current study.

## Abbreviations

CLBP: Chronic low back pain
SF-36: Short Form 36
VAS: Visual Analogic Scale
TUG: Timed Up and Go
